# Construction of a Six-Gene Prognostic Risk Model Related to Hypoxia and Angiogenesis for Cervical Cancer

**DOI:** 10.3389/fgene.2022.923263

**Published:** 2022-06-13

**Authors:** Lili Liu, Hongcang Zhu, Pei Wang, Suzhen Wu

**Affiliations:** ^1^ TCM Gynecology Department, Foshan Fosun Chancheng Hospital, Foshan Clinical Medical School of Guangzhou University of Chinese Medicine, Foshan, China; ^2^ Foshan Retirement Center for Retired Cadres, Guangdong Military Region of the PLA, Foshan, China; ^3^ Foshan Clinical Medical School, Guangzhou University of Chinese Medicine, Foshan, China

**Keywords:** cervical cancer, overall survival, hypoxia, angiogenesis, SsGSEA

## Abstract

**Background:** The prognosis of cervical cancer (CC) is poor and not accurately reflected by the primary tumor node metastasis staging system. Our study aimed to develop a novel survival-prediction model.

**Methods:** Hallmarks of CC were quantified using single-sample gene set enrichment analysis and univariate Cox proportional hazards analysis. We linked gene expression, hypoxia, and angiogenesis using weighted gene co-expression network analysis (WGCNA). Univariate and multivariate Cox regression was combined with the random forest algorithm to construct a prognostic model. We further evaluated the survival predictive power of the gene signature using Kaplan-Meier analysis and receiver operating characteristic (ROC) curves.

**Results:** Hypoxia and angiogenesis were the leading risk factors contributing to poor overall survival (OS) of patients with CC. We identified 109 candidate genes using WGCNA and univariate Cox regression. Our established prognostic model contained six genes (*MOCSI, PPP1R14A, ESM1, DES, ITGA5,* and *SERPINF1*). Kaplan-Meier analysis indicated that high-risk patients had worse OS (hazard ratio = 4.63, *p* < 0.001). Our model had high predictive power according to the ROC curve. The C-index indicated that the risk score was a better predictor of survival than other clinicopathological variables. Additionally, univariate and multivariate Cox regressions indicated that the risk score was the only independent risk factor for poor OS. The risk score was also an independent predictor in the validation set (GSE52903). Bivariate survival prediction suggested that patients exhibited poor prognosis if they had high z-scores for hypoxia or angiogenesis and high risk scores.

**Conclusions:** We established a six-gene survival prediction model associated with hypoxia and angiogenesis. This novel model accurately predicts survival and also provides potential therapeutic targets.

## Introduction

Cervical cancer (CC) is a malignant tumor that seriously threatens women’s health, ranking fourth in female-specific cancers (Cohen et al., 2019). In 2018, diagnosed cases reached over 560,000, and deaths numbered 300,000 (Bray et al., 2018). Although progress has been made in CC prevention, screening, and treatment (e.g., modern targeted technology and immunotherapy), the therapeutic effect remains insufficient (Li et al., 2016; Vu et al., 2018), even as annual incidence and associated mortality increase. Relapse and metastasis are major factors associated with CC-related deaths. However, the current tumor node metastasis staging system is ineffective in predicting patient prognosis. Therefore, a more efficient prognostic model or new prognostic markers are urgently needed to improve the clinical outcomes of patients with CC.

Recent applications of precision medicine and the advancement of second-generation sequencing have led to a growing number of studies that construct genomic models for cancer prognostic assessment (Cheng et al., 2019; Liu et al., 2021). Although some studies have established a prognostic model for CC, its limitations preclude widespread use in clinical practice (Chen H. et al., 2020; Chen et al., 2020c).

Previous studies have suggested that hypoxia in many cancers, including pancreatic cancer, neuroblastoma, gastric cancer, and bladder cancer, is closely related to poor prognosis (Chen et al., 2020b; Cangelosi et al., 2020; Jiang et al., 2021; Tao et al., 2021). The hypoxia risk model of glioma may reflect the strength of tumor immune response and independently predict prognosis. A hypoxia-related lncRNA signature and nomogram accurately predicted overall survival (OS) and disease-free survival of patients with gastric cancer (Chen et al., 2020b). Additionally, angiogenesis plays a critical role in tumor growth and metastasis, with data indicating a close connection to poor prognosis in lung adenocarcinoma, hepatocellular carcinoma, and breast cancer (Kerbel, 2008; Chen Y. et al., 2019; Teleanu et al., 2019; Korobeinikova et al., 2020; Yang et al., 2021). Angiogenesis-related genetic markers can effectively predict the prognosis of patients with gastric cancer, while angiogenesis-related gene-based nomograms allow for more precise risk stratification (Ren et al., 2020). However, the value of combining hypoxia- and angiogenesis-related gene expression in CC prognosis has rarely been investigated.

Therefore, our study aimed to establish a new prediction model for hypoxia and angiogenesis. First, through statistical analysis of data from The Cancer Genome Atlas (TCGA), we identified hypoxia and angiogenesis as two critical risk factors affecting the OS of patients with CC. We then established a gene signature related to hypoxia and angiogenesis and confirmed its predictive accuracy using a separate validation set from the Gene Expression Omnibus (GEO). Furthermore, we explored correlations between the risk model and immune infiltration.

## Materials and Methods

### Dataset Preparation and Data Processing

Clinical and transcriptome data from 257 patients with CC were collected from TCGA (http://cancergenome.nih.gov/) for use as training sets. A prognostic model was established from these data. The GSE52903 dataset from GEO (http://www.ncbi.nlm.nih.gov/geo/), containing transcriptome and clinical data of 54 patients with CC, was used as the validation set. As all data were downloaded from public databases, ethical approval was not required for this study.

### Candidate Gene Selection and Signature Establishment

Hallmark gene sets were downloaded from the Molecular Signatures Database (MSigDB) v.7.5.1 (https://www.gsea-msigdb.org/gsea/msigdb). Cancer hallmarks were assessed using single-sample gene set enrichment analysis (ssGSEA), implemented with R package “gsva” (Barbie et al., 2009; Liberzon et al., 2011). Hazard ratios (HR) of CC hallmarks were calculated using univariate Cox proportional hazards (Cox-PH) regression, implemented with R package “survival.” Based on ssGSEA scores and transcriptome data, a scale-free co-expression network was established using the weighted gene co-expression network analysis (WGCNA) R package to identify modules most related to hypoxia and angiogenesis (Langfelder and Horvath, 2008). Gene significance (GS) was calculated from correlations between individual genes and ssGSEA scores of hypoxia and angiogenesis. Associations between gene expression and module eigengenes were identified with module membership. Using the selection criteria of *p* < 0.0001 for GS and *p* < 0.01 for univariate Cox regression, 109 candidate genes were identified from the module that had the strongest association with hypoxia and angiogenesis. The importance of survival-related genes was ranked using the random forest algorithm. A Monte Carlo simulation with 100 iterations and 5 forward steps was performed (Ishwaran et al., 2008). The risk score model was established according to multivariate Cox regression using the following formula: risk score = β_1_x_1_+ β_2_x_2_+β_3_x_3_ + ⋯β_N_x_N_. Next, the best gene combination was selected based on log-rank *p* values obtained from Kaplan–Meier (KM) analysis.

### Survival Analysis Based on the Risk Model

Relationships between the best combination of genes and CC hallmarks were estimated using gene co-expression correlations (based on Pearson’s). Patients were classified into high- and low-risk groups with their median risk scores. Significant between-group differences in prognosis were determined using KM analysis. Prediction accuracy of the risk model was tested with a time-dependent receiver operating characteristic (tROC) curve and the area under the curve of the ROC (AUC) (Heagerty et al., 2000). Univariate and multivariate Cox regression models were used to evaluate the independent predictive values of each prognostic factor. The predictive accuracy of the risk model and individual prognostic factors was calculated using the concordance index (C-index) (Pencina and D'Agostino, 2004). Risk scores and hypoxia/angiogenesis hallmarks were combined for survival analyses and prognosis assessments in the training set.

### Correlation Analysis Between Risk Scores and Tumor Immune Microenvironment

Correlations between immune cell infiltration and risk scores were analyzed using the following analysis tools: TIMER, CIBERSORT, CIBERSORT-ABS, quanTIseq, MCPCOUNTER, EPIC, and xCELL. A heatmap was constructed to assess immune infiltration levels in high- and low-risk groups.

### Bioinformatics and Statistical Analysis

To identify CC-related genes, ssGSEA was performed using hypoxia and angiogenesis genomes from MSigDB (Subramanian et al., 2005). Data analysis and figure generation were conducted using R (version 4.1.1; https://www.r-project.org/). Both ssGSEA and risk scores were normalized using z-scores. Survival probability was assessed using KM analyses, and between-group differences in survival were determined using log-rank tests. Univariate and multivariate Cox regressions were performed to determine the effect of each factor on progression-free survival (PFS) and OS. The predictive capacity of risk models and cancer hallmarks were measured using tROC and AUC analyses (Heagerty et al., 2000), while risk-model prognostic accuracy was reflected in the C-index.

## Results

### Research Design

**FIGURE 1:**
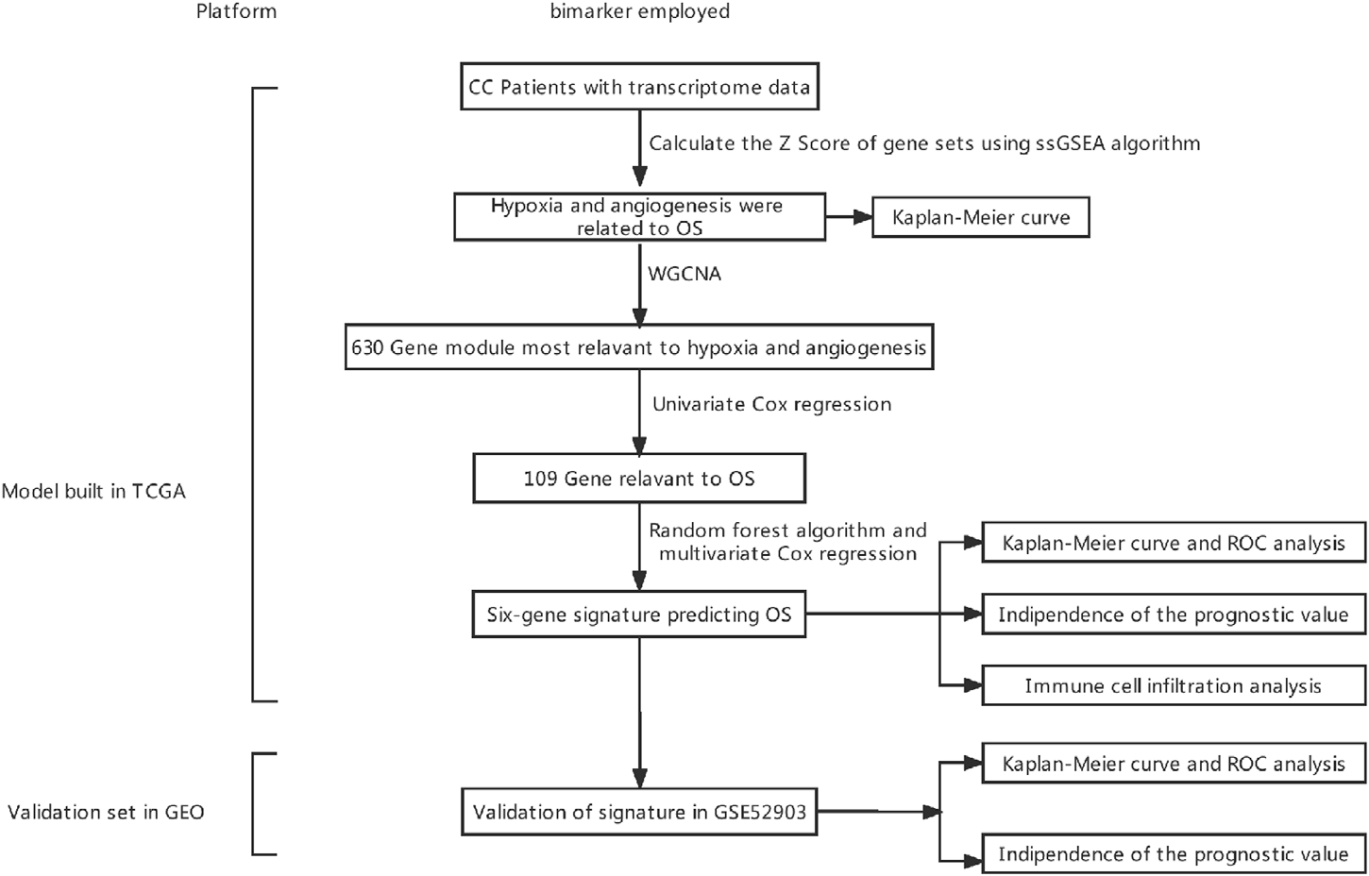
Experimental flowchart. CC, cervical cancer; WGCNA, weighted gene co-expression network analysis; ssGSEA, single-sample gene set enrichment analysis; OS, overall survival; ROC, receiver operating characteristic.

Figure 1 depicts the research protocol to generate a survival prediction model for patients with CC. We identified hypoxia and angiogenesis as the two cancer hallmarks most associated with OS. Next, we identified core hypoxia- and angiogenesis-related genes for survival prediction using a combination of WCGNA, univariate/multivariate Cox regression, and the random forest algorithm. We then used these core genes to build risk models for OS prediction. Finally, we assessed and validated the prognostic predictive power of the risk model in training and validation cohorts. Table 1 summarizes patient-related data.

**TABLE 1:** Clinical data from The Cancer Genome Atlas (TCGA) and Gene Expression Omnibus (GEO).

Characteristic	TCGA	GEO	*p*
n	257	54	
Status, n (%)			0.074
Alive	195 (62.7%)	34 (10.9%)	
Dead	62 (19.9%)	20 (6.4%)	
Stage, n (%)			0.141
Stage I	146 (46.9%)	27 (8.7%)	
Stage II	56 (18%)	8 (2.6%)	
Stage III	39 (12.5%)	15 (4.8%)	
Stage IV	16 (5.1%)	4 (1.3%)	
grade, n (%)			
G1	18 (7%)		
G2	127 (49.4%)		
G3	112 (43.6%)		
age, median (IQR)	46 (38, 56)	49 (41, 65.5)	0.220

### Hypoxia and Angiogenesis Were Key Hallmarks Affecting OS

**FIGURE 2:**
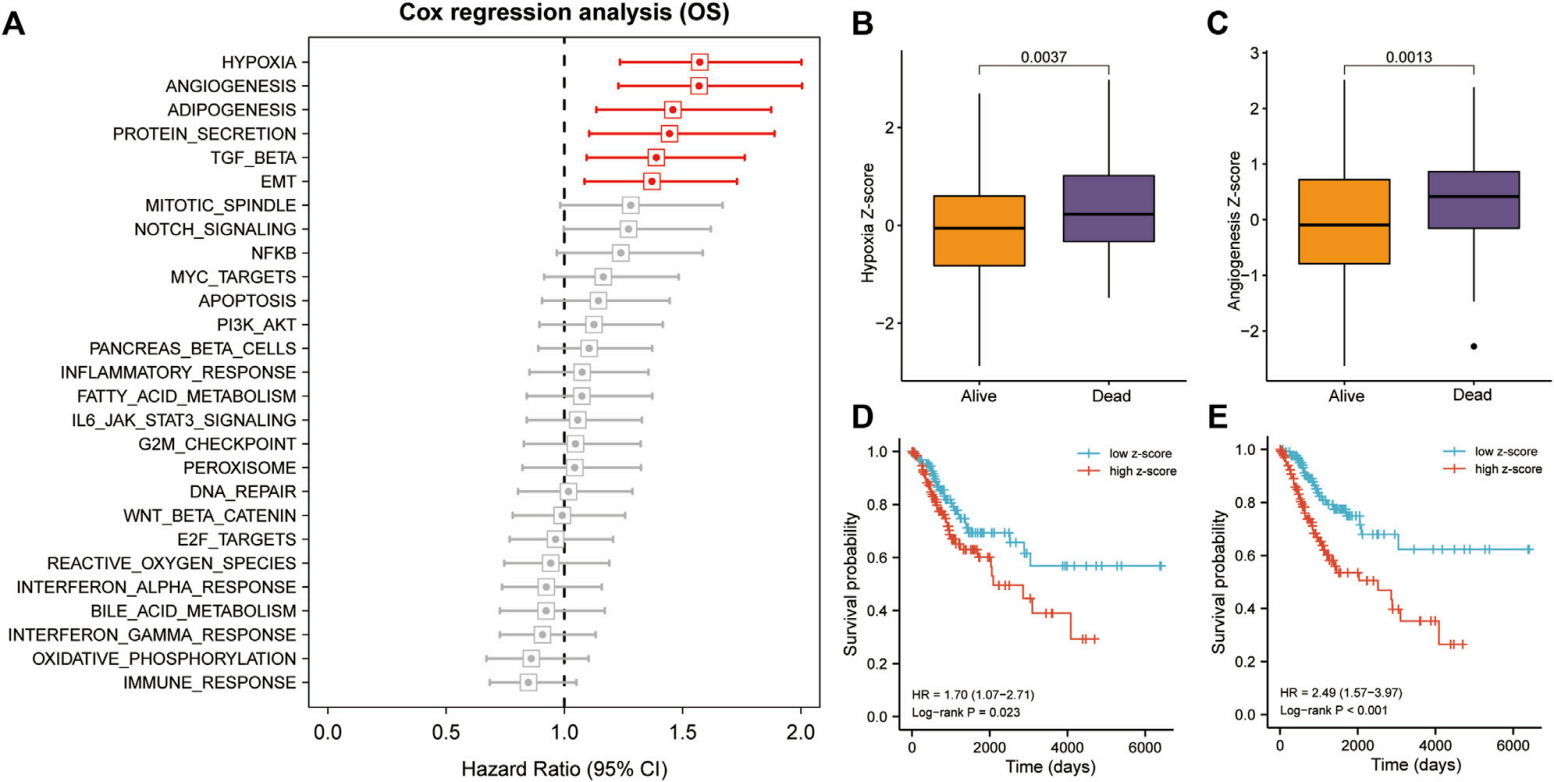
Hypoxia and angiogenesis were the key cancer hallmarks affecting OS in patients with CC. **(A)** Hypoxia and angiogenesis had a strong influence on OS, according to the univariate Cox-PH regression. **(B,C)** Patients with CC who died during follow-up had significantly higher z-scores for hypoxia and angiogenesis. **(D,E)** Patients with high hypoxia and angiogenesis z-scores had poorer OS, according to KM analysis. TGF, transforming growth factor; NFKB, nuclear factor kappa B; PI3k/Akt, phosphatidylinositol-3- kinase/serine-threonine kinase; EMT, epithelial-mesenchymal transition; KM, Kaplan–Meier.

We calculated and ranked Cox coefficients in terms of cancer-hallmark ssGSEA scores and the corresponding survival data of the training cohort. Univariate Cox-PH regression revealed that hypoxia and angiogenesis had a stronger influence on survival than adipogenesis, protein secretion, TGF-beta signaling, epithelial-mesenchymal transition, mitotic spindle, NOTCH signaling, NFKB, MYC-targets, apoptosis, PI3K/AKT signaling, pancreas-beta cells, or inflammatory response (Figure 2A). Hypoxia and angiogenesis z-scores were significantly higher in patients who died than in those who lived during the follow-up period (*p* < 0.05; Figures 2B,C). Using median risk scores, we assigned 257 patients with CC in the training cohort to high- and low-risk groups. Survival analysis indicated that patients with high hypoxia z-scores had poor OS (HR = 1.70, *p* = 0.023; Figure 2D), as did patients with high angiogenesis z-scores (HR = 2.49, *p* < 0.001; Figure 2E).

### Establishment of Prognostic Gene Signature Related to Hypoxia and Angiogenesis

**FIGURE 3:**
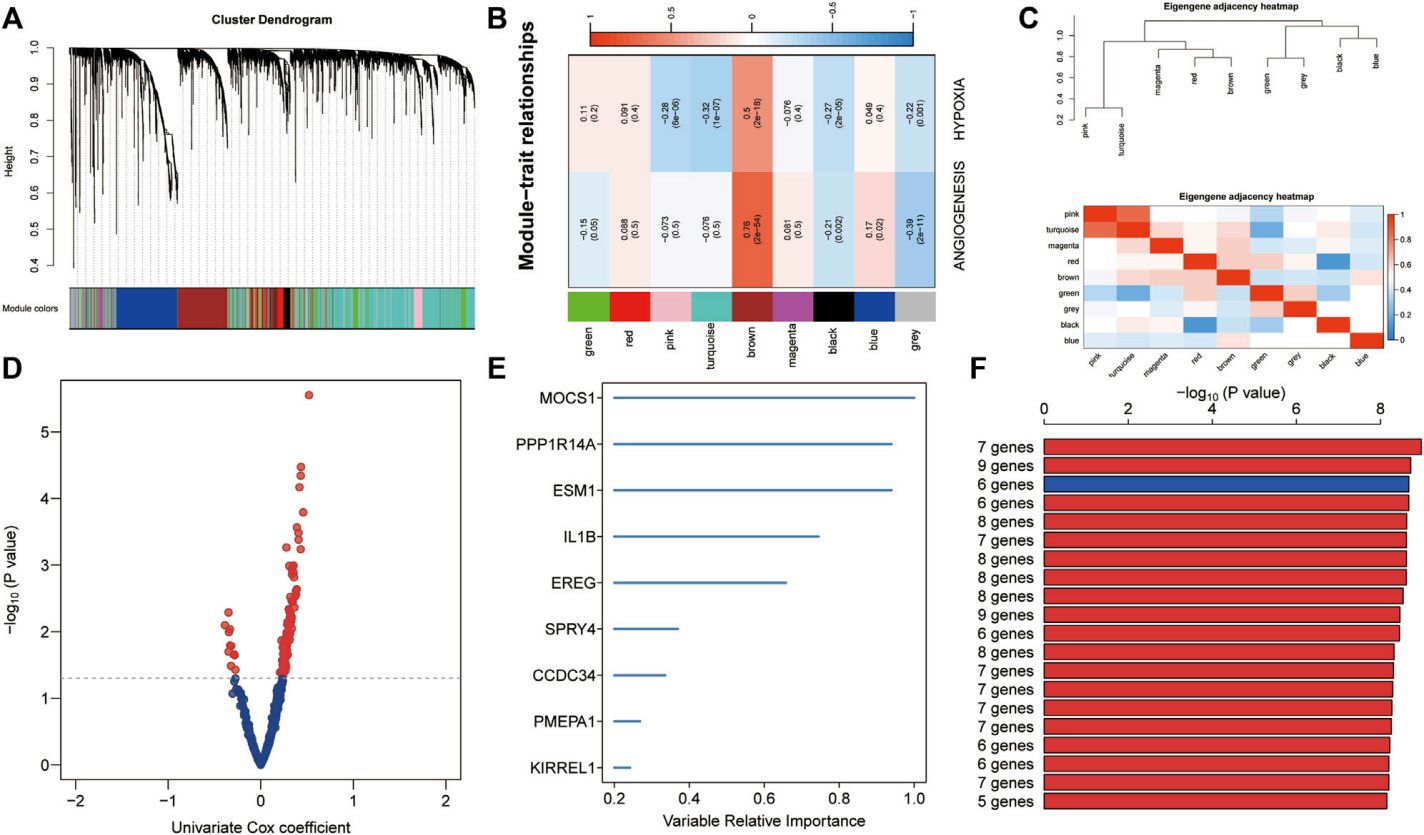
Establishment of hypoxia- and angiogenesis-related gene signatures. **(A)** Eight non-gray modules were confirmed using WGCNA. **(B)** The brown module was most significantly related to hypoxia and angiogenesis (r > 0.5, *p* < 0.0001). **(C)** Module eigengene adjacency heatmap displaying correlations of co-expressed modules. **(D)** Key genes from the brown module were screened. Univariate Cox regression was used to identify 109 candidate genes with prognostic potential. **(E)** The random forest supervised classification algorithm selected 10 genes from the 109 candidate genes. **(F)** The six-gene risk model was ranked first in KM analysis to identify optimal risk models.

To identify highly connected modules of co-expressed transcripts, we performed WGCNA using ssGSEA z-scores of hypoxia and angiogenesis from the training set and genome-wide microarray data (Figure 3A). Of the eight non-gray modules, the brown one was the most significantly related to hypoxia and angiogenesis (r > 0.5, *p* < 0.0001; Figure 3B). We displayed the correlation of co-expressed modules as a module eigengene adjacency heatmap (Figure 3C). We then used GS < 0.001 as the criterion for selecting key genes from the brown module. Univariate Cox regressions on these genes yielded 109 candidates with prognostic potential **(** *p* < 0.05; Figure 3D). From these candidate genes, the random forest supervised classification algorithm then extracted the top 10 (*EREG, ESM1, NAMPT, SERPINF1, PPP1R14A, MOCS1, ITGA5, NRP1, SPRY4,* and *DES*) (Figure 3E), forming 1024 risk model combinations. Using KM analysis and comparing -log_10_ P_log-rank_ values, we determined that the optimal risk model was the one with six genes (*MOCSI, PPP1R14A, ESM1, DES, ITGA5,* and *SERPINF1*). We considered that a good model should contain as few genes as possible (Figure 3F). The formula for establishing our model was as follows: risk score = 0.160 × *MOCSI* + 0.370 × *PPP1R14A* + 0.223 × *ESM1* + (-0.246) × *DES* + 0.323 × *ITGA5* + (-0.248) × *SERPINF1*.

### The Risk Score Is an Independent OS Predictor in the Training Set

**FIGURE 4:**
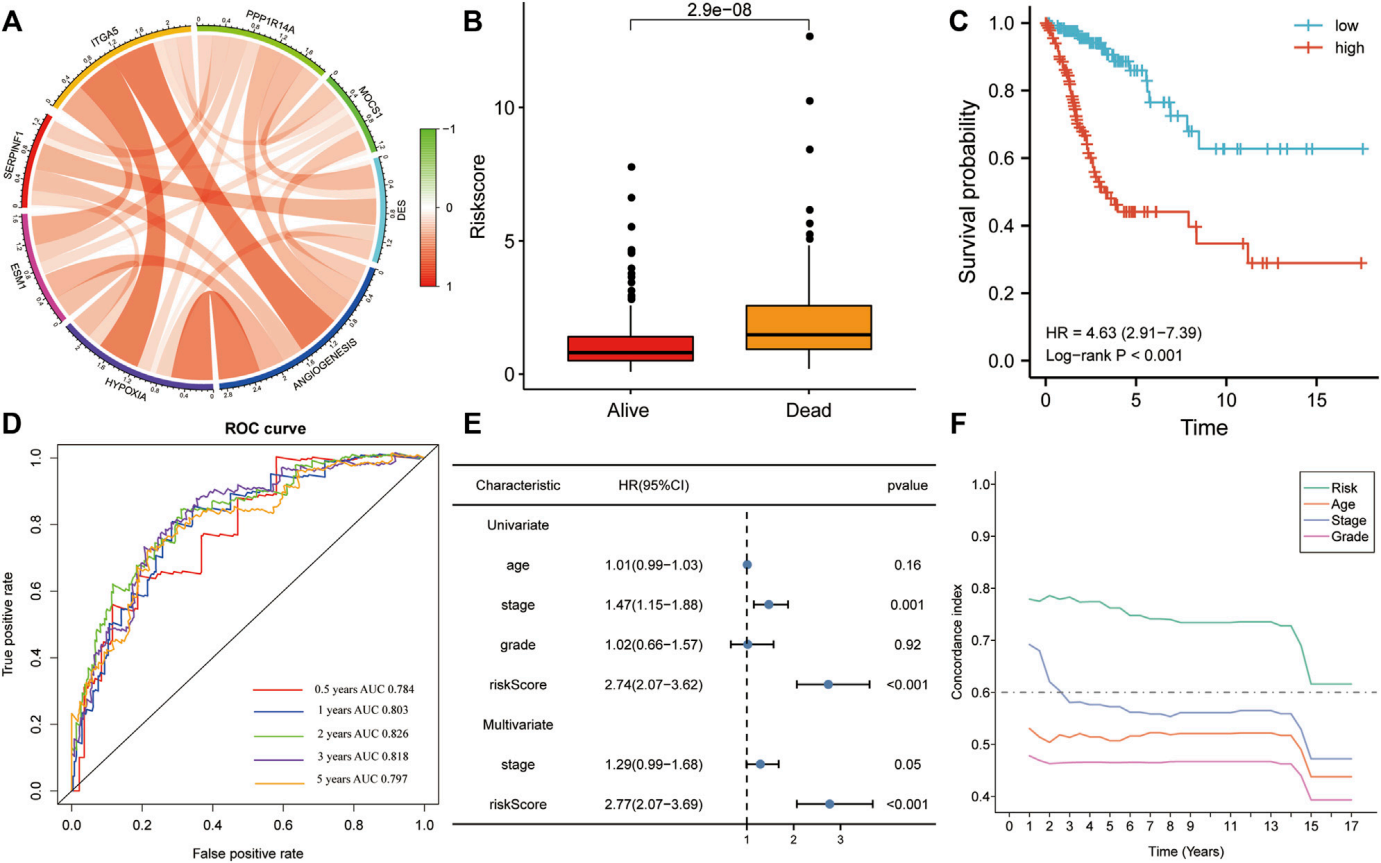
The risk score was the only significant independent risk factor affecting OS in the training set. **(A)** Correlation of the gene signature with hypoxia and angiogenesis**. (B)** The risk score was significantly higher in the mortality group during follow-up. **(C)** Patients with a high risk score had a poorer OS, according to KM analysis. **(D)** The risk score was a good predictor of OS (AUC > 0.7). **(E)** Univariate and multivariate Cox regression for OS revealed that risk score was the only significant independent risk factor among multiple clinicopathological variables. **(F)** The C-index indicated that the risk score had the best OS predictive ability among the clinicopathological variables. AUC, area under the ROC curve; HR, hazard ratio.

In the training set, all six genes were positively associated with hypoxia and angiogenesis (Figure 4A). Risk scores were significantly higher in the mortality group during follow-up (Figure 4B). The results of KM analysis for OS revealed that patients with high-risk scores had a poorer prognosis than those with low-risk scores (HR = 4.63, *p* < 0.001; Figure 4C). Additionally, the AUC-ROC analysis indicated that risk scores successfully predicted the 0.5-, 1-, 2-, 3-, and 5-year OS (AUC > 0.7; Figure 4D). Univariate and multivariate Cox regression for OS in the training set revealed that the risk score was the only significant independent risk factor among all tested clinicopathological variables (*p* < 0.001; Figure 4E). Moreover, the C-index indicated that the risk score had the best OS predictive ability (Figure 4F).

### The Risk Score Is an Independent PFS Predictor in the Training Set

**FIGURE 5:**
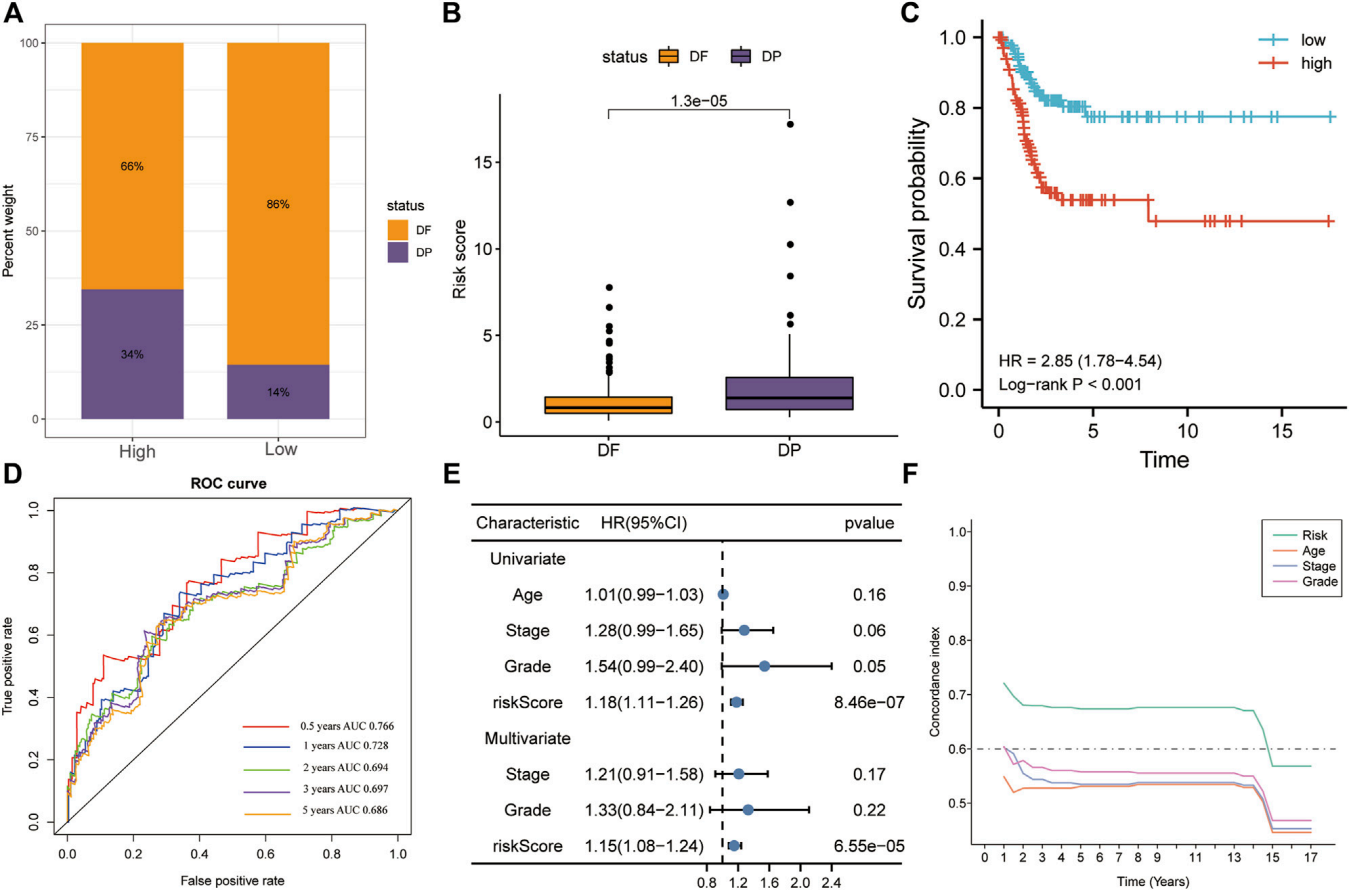
The risk score was the only significant independent risk factor predicting PFS in the training set. **(A)** The high-risk group contained a greater proportion of patients with disease progression. **(B)** Patients with disease progression had significantly higher risk scores. **(C)** Patients with high risk scores had a poorer prognosis, according to KM analysis. **(D)** The risk score was a good predictor of PFS (AUC > 0.68). **(E)** Univariate and multivariate Cox regression for PFS revealed that risk score was the only significant independent risk factor among included clinicopathological variables. **(F)** The C-index indicated that the risk score had the best PFS predictive ability. PFS, progression-free survival; AUC, area under the ROC curve.

The high-risk group had a greater proportion of patients with disease progression, whereas the low-risk group had a greater proportion of patients without progression (Figure 5A). During follow-up, risk scores were significantly higher in the disease-progression group than in the no-progression group (*p* < 0.01; Figure 5B). Patients with high risk scores had a poorer prognosis than those with low risk scores (KM analysis, HR = 2.85, *p* < 0.001; Figure 5C). The AUC-ROC analysis indicated that risk scores predicted 0.5-, 1-, 2-, 3-, and 5-year PFS (AUC > 0.68; Figure 5D). Like in OS, the risk score was the only significant independent risk factor for PFS (univariate/multivariate Cox regressions, *p* < 0.001; Figure 5E) and had the best predictive ability (Figure 5F).

**FIGURE 6:**
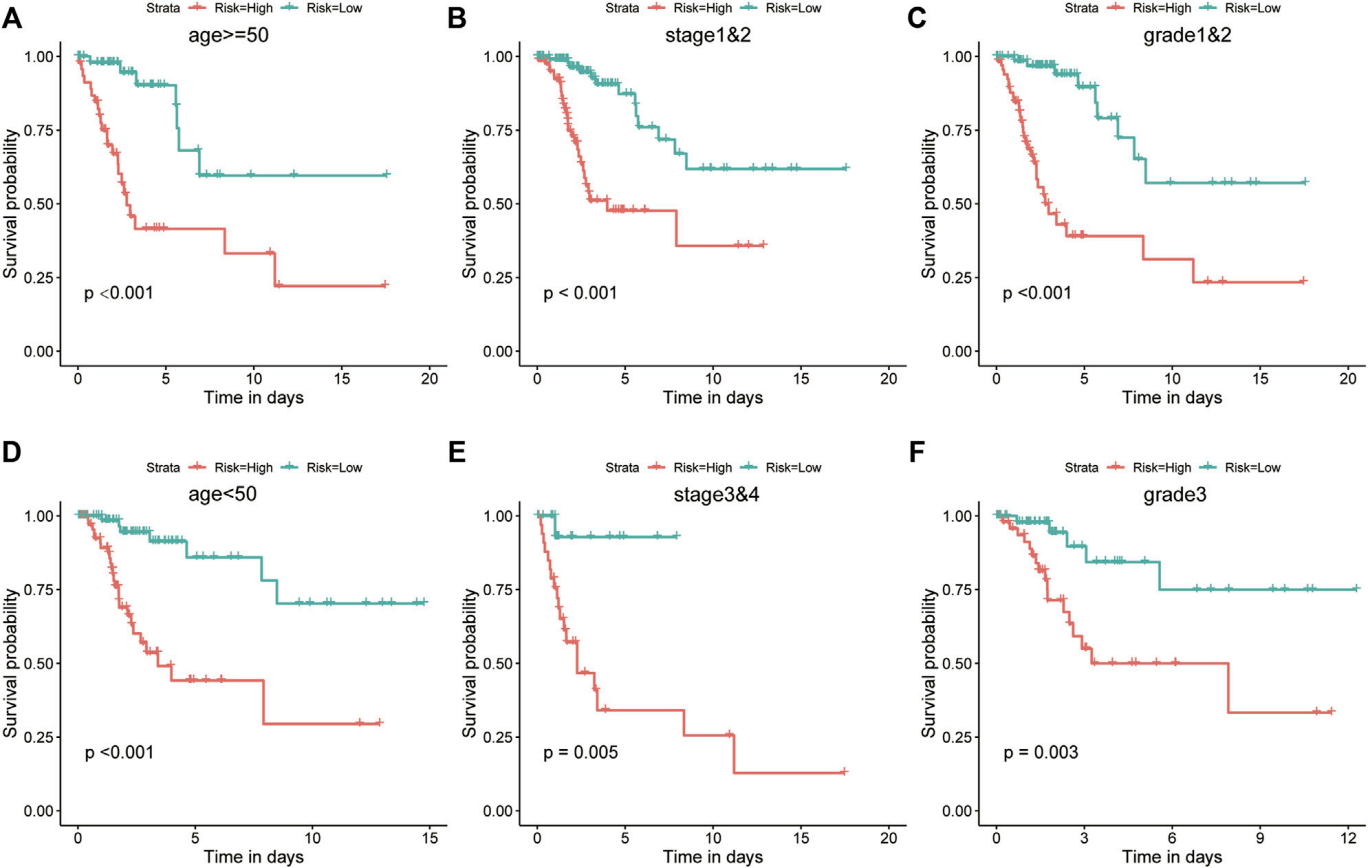
The risk score was a better predictor of OS across multiple subgroups of the training set. **(A–F)** KM analysis of OS was conducted for various clinicopathological subgroups, including age, stage, and grade. Patients with high risk scores had a worse prognosis.

In the training set, KM analysis of OS was conducted on patients after they had been divided into clinicopathological subgroups according to age, stage, and grade. Patients with high risk scores had a worse prognosis than those with low risk scores in all subgroups (Figures 6A–F).

### Risk Model Verification in the Validation Set

**FIGURE 7:**
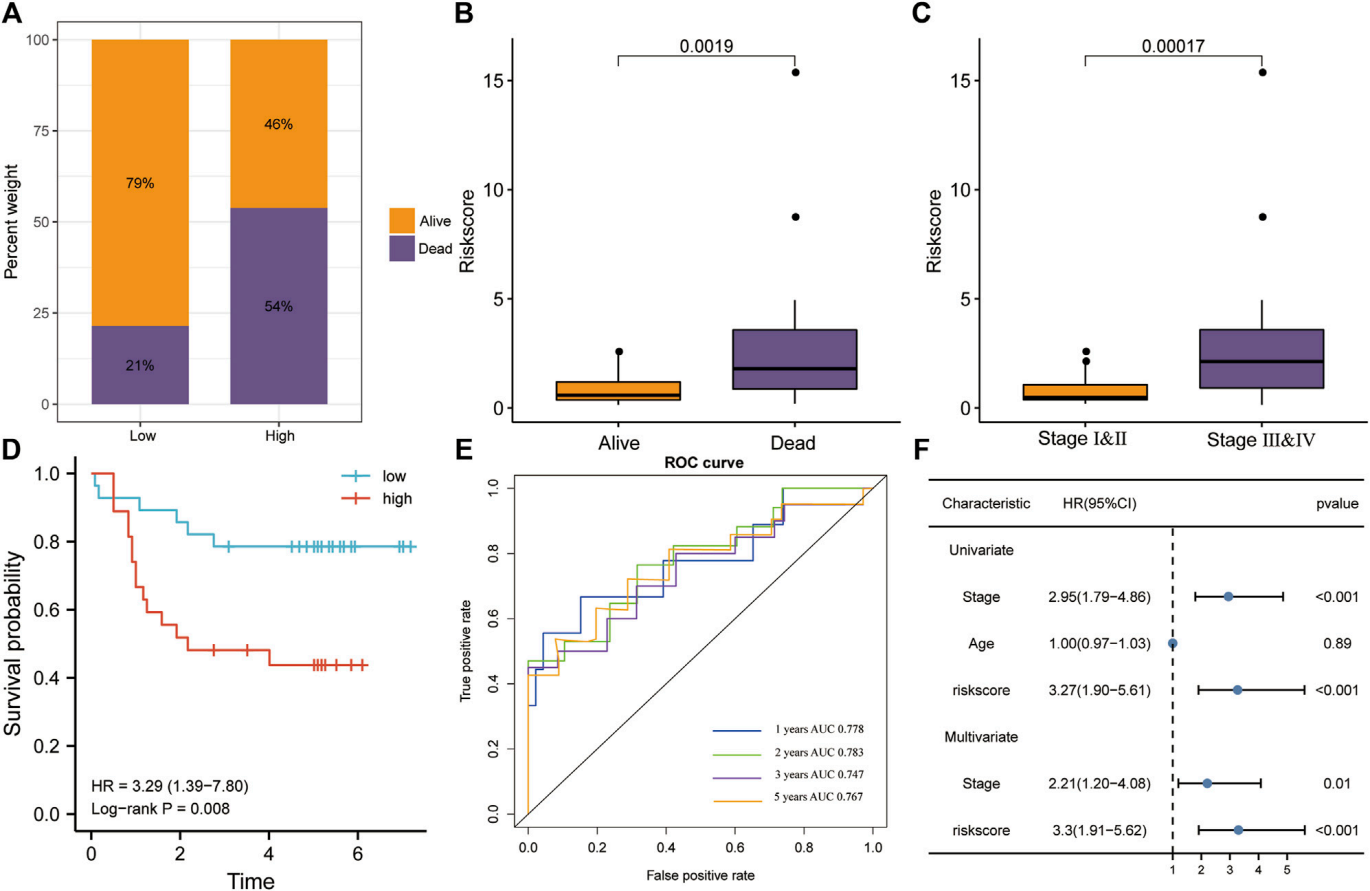
Verification of the risk model in the GSE52903 dataset. **(A)** The high-risk group had higher mortality rates. **(B)** The risk score was significantly higher in the mortality group during follow-up. **(C)** Patients in stages III–IV had significantly higher risk scores. **(D)** Patients with high risk scores had a poorer prognosis, according to KM analysis for OS. **(E)** The ROC analysis revealed that risk scores accurately predicted OS. **(F)** Univariate and multivariate Cox regression for OS revealed that risk score was the only significant independent risk factor among included clinicopathological variables.

We validated risk model performance using an independent CC validation set. The high-risk group had a greater proportion of patients who died, whereas the low-risk group had a greater proportion of surviving patients (Figure 7A). Risk scores were significantly higher in the mortality group during follow-up (*p* < 0.01; Figure 7B). Patients in stages III–IV had significantly higher risk scores than those in stages I–II (*p* < 0.001; Figure 7C). Patients with high-risk scores had a poorer prognosis in terms of OS than those with low-risk scores (KM analysis, HR = 3.29, *p* = 0.008; Figure 7D). Risk scores predicted the 1-, 2-, 3-, and 5-year OS in the validation set (AUC > 0.7; Figure 7E), while also being the only significant independent risk factor (univariate/multivariate Cox regressions, *p* < 0.001; Figure 7F).

Association and combined survival analysis of risk scores and key cancer hallmarks in the training set.

**FIGURE 8:**
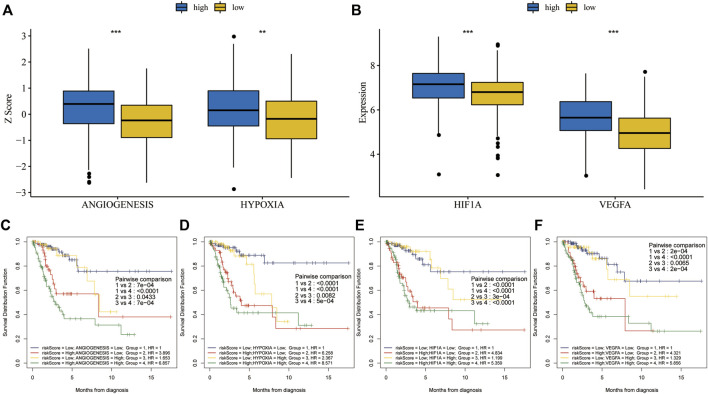
Combined survival analysis of risk scores and key cancer hallmarks in the training set. **(A)** Hypoxia and angiogenesis z-scores were significantly higher in the high-risk group. **(B)** *HIF1A* and *VEGFA* expression levels were significantly higher in the high-risk group. **(C,D)** The prognosis was worse in patients with high risk scores and high hypoxia or angiogenesis z-scores, according to KM analysis for OS after combining risk scores and key cancer hallmarks. **(E,F)** The prognosis was worse in patients with high risk scores and high HIF1A or VEGFA expression, based on KM analysis for OS after combining risk scores and genes related to key cancer hallmarks.

Hypoxia and angiogenesis z-scores were significantly higher in the high-risk group than in the low-risk group (Figure 8A). Vascular endothelial growth factor A (VEGFA) is a major driver of angiogenesis during tumor progression in various cancers (Krock et al., 2011). Hypoxia-induced factor 1 alpha (HIF1A) is a crucial protein in controlling hypoxia response (Li et al., 2020). HIF1A and VEGFA levels were significantly higher in the high-risk group than in the low-risk group (Figure 8B). When we ran KM analysis on combined risk scores and cancer hallmarks or hallmark-related genes, we found that OS prognosis was best with low risk scores and low hypoxia or angiogenesis z-scores (Figures 8C,D). Similarly, the prognosis was best with low risk scores and low HIF1A or VEGFA expression (Figures 8E,F).

### Relative Immune Infiltration Levels in High- and Low-Risk Groups

**FIGURE 9:**
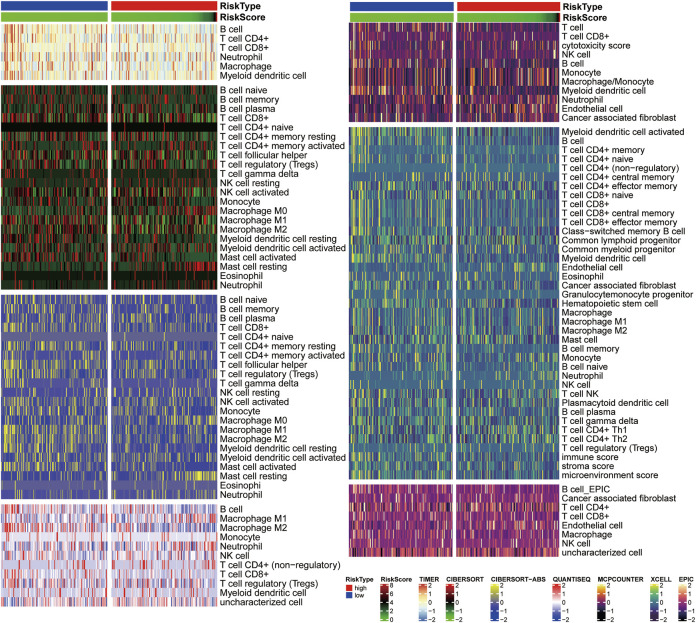
Correlation analysis of immune cell infiltration and risk scores. A Heatmap of seven different methods shows relative infiltration levels in high- and low-risk groups.

The heat map illustrates correlations between immune cell infiltration and risk scores (Figure 9). The TIMER analysis demonstrated that B cells and CD4+T cells were more abundant in the low-risk group. Additionally, CIBERSORT analysis showed that the low-risk group had more CD8+T cells, activating NK cells, M1 macrophages, M2 macrophages, and myeloid dendritic cells. The CIBERSORT-ABS analysis also found higher levels of CD8+T cells, CD4+T cells, follicular helper T cells, regulatory T cells, M1 macrophages, M2 macrophages, myeloid dendritic cells, and activated mast cells in the low-risk group. Both analyses revealed that resting mast cell and M0 macrophage levels were higher in the high-risk group. Furthermore, QuanTIseq analyses indicated that the low-risk group had more B cells, M2 macrophages, and CD8+T cells, whereas the high-risk group had more M1 macrophages and neutrophils. The low-risk group had more T cells, B cells, and myeloid dendritic cells, according to MCPCOUNTER analyses, whereas the high-risk group had more monocytes, macrophages, and endothelial cells. Along the same lines, EPIC analyses revealed that the numbers of CD8+T cells, CD4+T cells, B cells, myeloid dendritic cells, cancer-associated fibroblasts, and hematopoietic stem cells, and immune, stromal, and microenvironment scores were higher in the low-risk group. Finally, XCELL analyses suggested that B cell levels were higher in the low-risk group, while EPIC and XCELL analyses both found that endothelial cell levels were higher in the high-risk group.

## Discussion

The poor prognosis of CC remains a serious threat to women’s health, although human papillomavirus vaccination and screening have significantly reduced incidence and mortality. Early diagnosis and treatment are essential for improving CC prognosis, but reliable diagnostic and prognostic biomarkers are currently lacking.

Hypoxia and angiogenesis both predict poor prognosis in patients with CC (Vaupel and Mayer, 2007; Ding et al., 2019). Hypoxia involves insufficient oxygen supply to cells, tissues, or organs. Multiple studies have established its role in cancer tumorigenesis, development, invasion, metastasis, recurrence, and drug resistance (Rezaeian et al., 2017; Jing et al., 2019; Fico and Santamaria-Martinez, 2020). By promoting tumor angiogenesis, hypoxia facilitates rapid tumor growth, metastasis, and immune escape (Zhang, 2012; Dai et al., 2017). Unsurprisingly, a hypoxic microenvironment is closely associated with CC occurrence and development (Hockel et al., 1996). HIF-1α is activated in the hypoxic tumor microenvironment and modulates many transcription factors that allow cells to survive in unfavorable conditions (Semenza, 2012). Additionally, paclitaxel-resistant CC cells (HeLa-R cells) exhibit upregulated HIF1-α expression, and downregulation of HIF1-α re-sensitized HeLa-R cells to paclitaxel (Peng et al., 2014). Hypoxia-related genes appear to have latent prognostic value. For example, hCINAP (required for hypoxia-induced EMT and apoptosis) may play a role in CC metastasis and is a potential therapeutic target for CC (Zhang et al., 2021). Likewise, hypoxia-induced ZEB1 promotes CC progression via CCL8-dependent tumor-associated macrophage recruitment (Chen X.-J. et al., 2019).

Angiogenesis is the process of new vessel formation and a hallmark of solid tumors, including CC (Folkman, 1990). Substantial evidence has shown that angiogenesis contributes to CC development, progression, and metastasis (Kodama et al., 1999). Angiogenesis is important not only in tumor growth but also in hematogenous metastasis, as tumor blood vessels can provide nutrients and oxygen, while disposing metabolic waste. Angiogenesis is activated when the balance between stimulatory and inhibitory elements shifts towards pro-angiogenic factors (Hanahan and Folkman, 1996; Payen et al., 2015). One of the most important angiogenesis regulators is VEGF-A, a major proangiogenic cytokine in tumor growth and progression (Kerbel, 2008). VEGF-A also acts in CC (Cheng et al., 2000) and is associated with poor prognosis (Jin et al., 2017; Kaddu-Mulindwa et al., 2021).

Accumulating evidence suggests that hypoxia is closely associated with angiogenesis (Sui et al., 2017; Wen et al., 2019). Previous studies have revealed that HIF-1α regulates VEGFA expression *via* HIFα-dependent transcriptional activity (Manalo et al., 2005). Furthermore, antiangiogenic drugs help inhibit tumor growth and metastasis via the HIF-1α signaling pathway (Rey et al., 2017). To date, hypoxia-related prognostic gene signatures have been studied in prostate cancer (Yang et al., 2018), lung adenocarcinoma (Mo et al., 2020), and hepatocellular carcinoma (Zhang et al., 2020), whereas angiogenesis-related prognostic gene signatures have been studied in gastric cancer (Ren et al., 2020), renal clear cell carcinoma (Zheng et al., 2021), and breast cancer (Bender and Mac Gabhann, 2013). These findings revealed the prognostic value of hypoxia- and angiogenesis-related genes, along with their potential as therapeutic targets in CC.

However, these studies had certain flaws. First, hypoxia- or angiogenesis-related gene signatures were constructed based on considering individual genes reported in the literature, without considering that both processes likely involve entire gene networks. Second, such studies rarely investigated the prognosis predictive capacity of combining hypoxia-related gene signatures and angiogenesis-related gene signatures.

Therefore, our study established a new prognostic model based on gene signatures that correlate with hypoxia and angiogenesis. First, we applied ssGSEA and Cox-PH regressions to identify hypoxia and angiogenesis as cancer hallmarks most significantly associated with OS in patients with CC. Because hypoxia can promote angiogenesis, the two phenotypes are strongly correlated. Subsequently, we used WGCNA to identify the gene module most strongly associated with both processes. We then obtained prognostic hub genes (including *MOCS1, PPP1R14A, ESM1, DES, ITGA5,* and *SERPINF1*) after univariate Cox regression, random forest algorithm, and KM analysis. This method allowed us to comprehensively identify genes associated with both phenotypes, given that the regulation of hypoxia and angiogenesis occurs in a network. These analyses will enhance our understanding of hypoxia and angiogenesis regulatory mechanisms. Next, survival analysis of training and validation sets demonstrated that our six-gene prognostic model independently predicted OS in patients with CC. Finally, immune cell infiltration analysis suggested that high-risk patients had significantly lower infiltration levels.

Each of the six genes have been implicated in cancer. *PPP1R14A* is involved in the pathogenesis of human melanoma. It drives Ras activity and tumorigenesis by activating the growth-promoting *ERM* family and inhibiting the tumor suppressor merlin (Riecken et al., 2016). *ESM1* has been widely explored in various cancers, including prostate cancer, hepatocellular carcinoma, and head and neck squamous cell carcinoma; it also has prognostic value in esophageal cancer (Calderaro et al., 2019; Xu et al., 2019; Cui et al., 2021; Pan et al., 2021). *ITGA6* is an oncogene in various cancers (Raab-Westphal et al., 2017), including CC, where it is overexpressed and associated with proliferation and invasion (Yang J. et al., 2019). In contrast to these genes, *MOCS1*, *DES*, and *SERPINF1* are poorly understood. Thus, their potential biological functions require further research.

Dysfunction of the antitumor immune system is closely related to CC development and progression (Chen Z. et al., 2019; Liu J. J. et al., 2020; Liu X. et al., 2020). High levels of activated memory CD4+T cells predict a better prognosis in patients with CC (Wang et al., 2019). The correlation between risk stratification and immune cell infiltration further demonstrates the predictive power of our six-gene prognostic model.

Previously, a nine-lncRNA signature was established to predict the 1 year PFS in patients with CC; this model had an AUC of 0.793, 0.780, and 0.742 in two GEO test sets and one TCGA test set, respectively (Mao et al., 2019). A seven-gene prognostic signature for CC had also been developed using GEO data, predicting 1-, 3-, and 5-year OS with AUC of 0.74, 0.76, and 0.81, respectively (An et al., 2022). Another study established 11 immune-related gene signatures to assess OS in patients with CC, yielding 3- and 5-year AUC of 0.733 and 0.747 (Yang S. et al., 2019). For our six-gene prognostic risk model, the AUC at 1, 2, 3, and 5 years is 0.784, 0.803, 0.826, 0.818, and 0.797 in the training set, and 0.778, 0.783, 0.747, and 0.767 in the validation set. Compared to the previous models, ours showed better predictive power.

Nevertheless, our study had some limitations. First, our findings would be better supported with the inclusion of more machine learning tools. The random forest algorithm is a mature and widely used machine learning method, with relatively stable results. However, new machine learning methods are available that can benefit our investigation, including a novel tool for gene selection and phenotype classification, as well as an efficient algorithm for survival analysis and biomarker selection (Huang et al., 2021; Huang et al., 2022). Using these more advanced techniques should reduce errors from platforms or samples. Another limitation was that we only used one GEO dataset for verification and did not include normal transcript data as a control. Therefore, future studies need to validate the predictive value of our six-gene signature in more CC tissues and adjacent normal tissues. Finally, we still know little about the biological functions of the six hypoxia- and angiogenesis-related genes, necessitating more experiments in the future.

## Conclusion

In summary, we established a new six-gene signature for CC and used it to develop a risk model that strengthens prognostic predictions. The six-gene prognostic model should be an effective tool for detecting high-risk patients, enabling early treatment to maximally prevent CC advancement. While possessing high predictive power, this model also has a small number of genes, reducing the economic burden on patients. Thus, it has great potential for clinical application and transformation. The genes chosen for the model play a very important role in tumor development, suggesting that they can be potential therapeutic targets. Our model is not only useful for predicting prognosis, but can also supplement the existing TNM staging method. Once the model is verified in more clinical cases, our data can be generalized to a larger population.

## Data Availability

Publicly available datasets were analyzed in this study. This data can be found here: Clinical and transcriptome data were obtained from the TCGA (http://cancergenome.nih.gov/). The GSE52903 dataset was obtained from GEO (http://www.ncbi.nlm.nih.gov/geo/).
